# 

**Published:** 1893-01

**Authors:** 


VOL. XIV. JANUARY, 1893. No. 1.

Jan. 18

LIBRARY Index S. G. O.

### THE

### International Dental Journal

128

A MONTHLY PERIODICAL

DEVOTED TO

### DENTAL AND ORAL SCIENCE.

JAMES TRUMAN, D.D.S., EDITOR.

JOSEPH HEAD, M.D., D.D.S., ASSISTANT EDITOR.

Publication Office,

716 FILBERT STREET, PHILADELPHIA.

SURGEON GEN'L'S OFFICE. LIBRARY.

PUBLISHED BY THE

INTERNATIONAL DENTAL PUBLICATION COMPANY,

51 WEST THIRTY-SEVENTH STREET, NEW YORK CITY,

—AND—

716 FILBERT STREET, PHILADELPHIA.

HARRY T. PEARCE, ADVERTISING MANAGER, P. O. BOX 151, PHILADELPHIA, PA.

Entered at the Post-Office at Philadelphia, and admitted for transmission through the mails at second-class rates.

Subscriptions must begin with the January or July number.

$2.50 per Annum, in Advance. Single Copies, 25 Cents.

PRINTED BY J. B. LIPPINCOTT COMPANY, PHILADELPHIA.

